# Transcallosal white matter and cortical gray matter variations in autistic adults ages 30-73 years: A bi-tensor free water imaging approach

**DOI:** 10.21203/rs.3.rs-4907999/v1

**Published:** 2024-08-16

**Authors:** Young Seon Shin, Danielle Christensen, Jingying Wang, Desirae J. Shirley, Ann-Marie Orlando, Regilda A. Romero, Bradley J. Wilkes, David E. Vaillancourt, Stephen Coombes, Zheng Wang

## Abstract

**Background:** Autism spectrum disorder (ASD) has long been recognized as a lifelong condition, but brain aging studies in autistic adults aged >30 years are limited. Free water, a novel brain imaging marker derived from diffusion MRI (dMRI), has shown promise in differentiating typical and pathological aging and monitoring brain degeneration. We aimed to examine free water and free water corrected dMRI measures to assess white and gray matter microstructure and their associations with age in autistic adults. **Methods:** Forty-three autistic adults ages 30-73 years and 43 age, sex, and IQ matched neurotypical controls participated in this cross-sectional study. We quantified fractional anisotropy (FA), free water, and free water-corrected FA (fwcFA) across 32 transcallosal white matter tracts and 94 gray matter areas in autistic adults and neurotypical controls. Follow-up analyses assessed age effect on dMRI metrics of the whole brain for both groups and the relationship between dMRI metrics and clinical measures of ASD in regions that significantly differentiated autistic adults from controls. **Results:** We found globally elevated free water in 24 transcallosal tracts in autistic adults. We identified negligible differences in dMRI metrics in gray matter between the two groups. Age-associated FA reductions and free water increases were featured in neurotypical controls; however, this brain aging profile was largely absent in autistic adults. Additionally, greater autism quotient (AQ) total raw score was associated with increased free water in the inferior frontal gyrus pars orbitalis and lateral orbital gyrus in autistic adults. **Limitations:** All autistic adults were cognitively capable individuals, minimizing the generalizability of the research findings across the spectrum. This study also involved a cross-sectional design, which limited inferences about the longitudinal microstructural changes of white and gray matter in ASD. **Conclusions:** We identified differential microstructural configurations between white and gray matter in autistic adults and that autistic individuals present more heterogeneous brain aging profiles compared to controls. Our clinical correlation analysis offered new evidence that elevated free water in some localized white matter tracts may critically contribute to autistic traits in ASD. Our findings underscored the importance of quantifying free water in dMRI studies of ASD.

## INTRODUCTION

Autism spectrum disorder (ASD) is a lifelong condition that profoundly impacts health, independence, and quality of life [1–4]. Previous studies of autistic children have identified corpus callosum as a mostly implicated white matter contributing to critical clinical features of ASD (for a review, see [5]). Reduced fractional anisotropy (FA) and increased mean diffusivity (MD) in the corpus callosum have been found in several diffusion MRI (dMRI) meta-analysis studies of autistic children, highlighting premature inter-hemispheric integrity and myelin formation during early development [6–8]. These findings, combined with evidence demonstrating reduced structural connectivity in long-range intra-hemispheric tracts, have led to the underconnectivity hypothesis in ASD [9, 10]. Currently, little is known about microstructural variations in the brain of autistic adults aged 30 years and above. The sparsity of structural imaging research in autistic adults hampers our comprehensive understanding of brain development in ASD across the adult lifespan.

Diffusion MRI has advanced our understanding of axonal architecture and neuronal integrity in vivo [11–15]. Conventional dMRI analysis being used in most studies of ASD is performed by modeling a single diffusion tensor in each voxel. However, single tensor modeling suffers from free water-induced fractional volume contamination and leads to biased scalar metric estimation. Free water consists of freely moving water molecules without directional restriction [16, 17]. Free water typically comprises cerebrospinal fluid enclosed within the ventricles and surrounding the brain parenchyma [17–19]. As individuals age, free water accumulates in the extracellular space due to neuroinflammation, cytotoxicity, neuronal degeneration, and axonal shrinkage, making it a sensitive marker that monitors pathological aging disease progression [17, 20].

To account for partial volume effects induced by free water, a bi-tensor approach can be applied to dMRI analysis, which estimate extracellular free water and remove fractional volume contamination to offer more precise estimates of dMRI scalar metrics in brain tissue [17–19]. Together, these measures of free water and the corrected scalar metrics (e.g. free water corrected FA - fwcFA) provide a more comprehensive and nuanced microstructural evaluation of the brain. This approach is critical to examining microscopic variations in the brain of autistic individuals because they may be at greater risk for elevated free water compared to neurotypical controls. For example, postmortem brain studies in autistic children have demonstrated aberrant myelination, reduced minicolumn width, and enlarged ventricles [21–25]. These findings may be associated with increased extracellular space and thus greater free water accumulation during early development. Besides, brain aging is typically accompanied by increased free water and reduced FA, adding an additional layer of vulnerability for autistic adults [11–13].

Localized elevations of free water have been observed in two studies of ASD. One large-cohort study identified free water increases in multiple cortical-basal ganglia tracts in autistic children ages 7–18 years, and elevated free water in bilateral dorsolateral prefrontal cortex to caudate tract was significantly associated with increased restricted and repetitive behaviors in ASD [26]. Another study found elevated free water in the hippocampus of autistic adults ages 40–66 years, which was additionally correlated with a steeper decline of long-term visual memory loss over a 2–4 year span [27]. These seminal findings demonstrate that free water is elevated in specific regions in the brain across the ASD lifespan. In the current study we advance the literature by assessing free water across the whole brain in autistic adults, including transcallosal white matter tracts and cortical and subcortical gray matter areas.

Employing a bi-tensor model to our dMRI analysis, we quantified free water and free water-corrected FA (fwcFA) across 32 transcallosal white matter tracts and 94 gray matter regions of interest (ROIs) in autistic adults and neurotypical controls. For comparison, we also employed traditional single tensor modeling to estimate FA (uncorrected) in the same ROIs. We hypothesized that autistic adults would show negligible FA and fwcFA variations but increased free water in the transcallosal white matter when compared to neurotypical controls because limited existing literature has observed non-significant FA, MD, and radial diffusivity (RD) in the corpus callosum (genu) in middle and old aged autistic adults relative to controls [28]. We also extend this hypothesis to gray matter, despite a limited number of studies identifying contradictory results of negligible [29] or different [30] gray matter structural variations in middle and old aged autistic adults compared to controls. Additionally, we examined age effects for white and gray matter between autistic adults and neurotypical controls, predicting that the autistic group would exhibit more pronounced FA and fwcFA reductions but free water increases respective to age, consistent with previous work in autistic adults at similar ages [26, 27, 29, 31]. Finally, we examined the relationship between dMRI metrics and clinical measures of ASD in regions that significantly differentiated autistic adults from neurotypical controls.

## MATERIALS AND METHODS

All procedures involved in this study were approved by the University of Florida (UF) Institutional Review Board following the Declaration of Helsinki. The IRB number is 202100659, with an approval date of July 26, 2022.

## Study Participants

Forty-three autistic adults and forty-three neurotypical controls participated in this study. Participants were between 30 and 73 years old and groups were matched on age, sex, and intelligence quotient (IQ) (Table 1). Autistic adults were identified and recruited from the Center for Autism and Related Disabilities (CARD) at the University of Florida in Gainesville, the University of Central Florida, the University of South Florida, and the SPARK Research Match. Neurotypical controls were recruited primarily from communities in north central Florida through study flyers and word of mouth. All participants provided written informed consent after receiving a complete description of the study. All participants completed the Repetitive Behavior Scale-Revised (RBS-R) [32] and had their IQ assessed using the Wechsler Abbreviated Scales of Intelligence, 2nd Edition (WASI-II) [33].

Table 1
Demographic and clinical characteristics between autistic adults (ASD) and neurotypical controls (NT)

| | ASD Mean (± SD) | NT Mean (± SD) | $t/\chi^2$ | p |
|---|---|---|---|---|
| **Age (years)** | 47.21 (± 10.86) | 49.79 (± 12.01) | -1.05 | 0.299 |
| Range | 30–73 | 30–70 | | |
| **Sex (M/F)** [b] | 25/18 | 23/20 | 0.19 | 0.664 [a] |
| **Handedness (R/L/B)** [a/b] | 39/3/1 | 39/4/0 | 1.14 | 0.565 [a] |
| **Full-scale IQ** | 107.44 (± 13.71) | 107.87 (± 11.02) | -0.16 | 0.877 |
| **Verbal IQ** | 107.72 (± 13.78) | 106.21 (± 11.23) | 0.54 | 0.589 |
| **Non-verbal IQ** | 105.33 (± 13.90) | 107.90 (± 13.49) | -0.85 | 0.399 |
| **AQ** | 34.72 (± 7.25) | 13.84 (± 5.64) | 14.07 | **< .001*****|
| **SRS-2** | 76.47 (± 7.91) | 45.23 (± 3.96) | 9.93 | **< .001***** |
| **ADOS-2** | 10.72 (± 3.31) | n/a | | |
| **RBS-R** | 44.44 (± 26.88) | 3.28 (± 3.73) | 21.21 | **< .001***** |
| **Total brain volume ($cm^3$)** | 1555.34 (± 161.11) | 1515.17 (± 146.34) | 1.21 | 0.230 |

[a] Self-reported handedness: R = right-hand dominant, L = left-hand dominant, and B = ambidextrous

[b] Chi-square ($\chi^2$) statistics

Total raw scores were reported for ADOS-2, AQ, and RBS-R; *t* scores were applied for SRS-2

Statistical significance is in bold-faced. *p < 0.05, **p < 0.01, ***p < 0.001

**Supplementary materials**

[Insert Supplementary Fig. 1 about here]

**Supplementary Fig. 1. b** dispersion plots for 32 transcallosal tracts (top panel) and 94 gray matter ROIs (bottom panel) of autistic adults (rose red) and neurotypical controls (sky blue). Dispersion plots for FA (A), free water (B), and fwcFA (C) are arranged from the left to the right. The that sits in the middle of each dispersion plot represents the [Mean ± SE] of **b** value for each autism or control group. The [Mean]s of **b** values derived from each of the frontal, temporal, parietal, and occipital cortices are displayed at the bottom panel of each dispersion plot cluster and labeled by. The lines connecting these red and blue dots represent the **b** mean difference between the autism and control groups.

[Insert Table 1 about here]

Prospective autistic adults with a clinical diagnosis of ASD were screened using the Autism Spectrum Quotient for Adults (AQ) [34] and the Social Responsiveness Scale Adult Self-Report (SRS-2) [35]. The AQ comprises five sub-scales that evaluate individuals' social skill, attention switching, attention to detail, communication skill, and imagination. The SRS-2 includes sub-scale assessments on social awareness, social cognition, social communication, social motivation, restricted interest, and repetitive behavior. Individuals who scored $>$ 32 on the AQ and $\geq$ 65 on the SRS-2 were invited to receive a diagnostic evaluation using the Autism Diagnostic Observation Schedule, Second Edition (ADOS-2) [36] at the UF CARD. Diagnosis for autistic adults was confirmed through a comprehensive review of AQ, SRS-2, ADOS-2, and expert clinical opinion following the DSM-5 criteria [37]. Three autistic adults did not meet the cut-off for AQ or SRS-2 but scored > 7 on ADOS-2. Their diagnosis was later confirmed by research reliable clinicians (AMO and RAR) on our team. Autistic adults were excluded if they had a known genetic or metabolic disorder associated with ASD (e.g., Fragile X syndrome, Rett syndrome, Phelan McDermid syndrome, tuberous sclerosis).

Prospective controls who scored $\leq$ 22 on the AQ and < 60 on the SRS-2 were recruited to the study. Prospective controls were excluded if they reported a family history of ASD or other neurodevelopmental disorders in their first- and second-degree relatives. Prospective autistic adults and controls who met any of the following criteria were excluded from the present study: 1) confirmed diagnosis of intellectual disability, mild cognitive impairment, or dementia; 2) confirmed diagnosis of non-specific developmental delay; 3) recent history of or current major psychiatric conditions (e.g., schizophrenia, bipolar disorder or post-traumatic stress disorder); 4) recent history of or current medical illness that significantly affects the structure and/or function of the central nervous system (e.g., brain tumor, thyroid disease, Cushing's disease, or HIV infection); 5) confirmed diagnosis of a neurological disorder (e.g., stroke, dystonia, seizure disorders, Parkinson's disease, or cerebellar ataxia); 6) family history of a hereditary neurological disorder (e.g., Huntington's Chorea, Wilson's Disease, or amyotrophic lateral sclerosis); 7) substance use disorder within six months prior to testing or a significant long-term history of substance use disorder; 8) wearing implanted medical devices (e.g., pumps, cardiac pacemakers, or cochlear implants); 9) pregnant; 10) had a full-scale IQ (fs-IQ) < 75, or 11) non-English speaking.

Lastly, one autistic adult reported birth asphyxia, and two autistic adults reported prolonged delivery at birth. Nine autistic adults reported a history of concussion due to risky play during childhood or car collisions during adulthood. Medication being used within 48 hours prior to testing included antipsychotics (ASD = 4), mood stabilizers (ASD = 2), stimulants (ASD = 7), antidepressants (ASD = 26, NT = 4), and sedatives (ASD = 6, NT = 2).

## dMRI data acquisition

The MRI session was administered on a 3T Siemens Prisma scanner with a 64-channel head coil at the UF McKnight Brain Institute. dMRI images were acquired using an echo-planar imaging sequence with the following parameters: TR = 6400 ms, TE = 58 ms, voxel size = 2.0 mm x 2.0 mm x 2.0 mm, b-values: 5 × 0, and 64 × 1,000 s/mm$^2$, field of view = 256 x 256, number of continuous slices = 69, and bandwidth = 2442 Hx/pixel. Participants wore earplugs and headphones to minimize discomfort from instrumental noise. Head motion was restricted using foam paddings inserted around the head. The scan took about 7 minutes and 41 seconds to complete.

### dMRI data post-processing and analysis

All dMRI data underwent post-processing and analysis using FMRIB Software Library 6.0 (FSL, fsl.fmrib.ox.ac.uk; [38, 39]). dMRI data were corrected for eddy current induced distortions and head motion using a three-dimensional (3D) affine transformation for all participants. Gradient directions were then rotated to reflect these corrections, and brain data were extracted afterward [40, 41]. A diffusion tensor model was fit to the eddy and motion corrected data to determine voxel-wise FA. Consistent with prior work from our group and others [15, 17, 42], we calculated a whole brain free water map for each individual to estimate the fractional volume of freely diffusing water in each voxel using custom MATLAB scripts (R2023a, The Mathworks, Natick, MA, USA). The free water map was then applied to correct the FA map, leading to a free water corrected FA (fwcFA) map. All images were registered to in-house templates via a nonlinear warping procedure using the SyNCC option in the Advanced Normalization Tools (ANTs) [43]. The registration procedure applied both an affine and deformation transformation to the whole brain maps using cross correlation as the optimization metric. Whole brain FA, free water, and fwcFA maps were transformed to Montreal Neurological Institute (MNI) 152 standard space (1 mm isotropic). After artifact inspection, mean diffusion metrics were derived from these maps for white and gray matter.

We extracted the mean of FA, free water, and fwcFA from white matter using the transcallosal tractography template (TCATT; [42]) and gray matter using the Mayo Clinic Adult Lifespan Template (MCALT; [44]). The TCATT is an ROI-based template that consists of 32 commissural tracts between homotopic regions of both hemispheres in 3D. This template includes transcallosal tracts from the frontal (17), temporal (3), parietal (6), and occipital (6) cortices [42]. Using an innovative slice-level thresholding approach, TCATT advances the spatial resolution of transcallosal tracts and reduces the likelihood of false positives relative to conventional templates [42]. The MCALT was constructed from T1-weighted scans of 202 healthy controls aged > 30 years [44] making it suitable for our study given the wide age range. The MCALT includes 94 ROIs from the frontal (36), temporal (22), parietal (16), occipital (12) cortices and subcortical (8) regions. The mean values of FA, free water and fwcFA were derived for each transcallosal tract and gray matter ROI, totaling 378 [(32 transcallosal tracts + 94 ROIs) $\times$ 3 diffusion measures = 378] dependent variables.

### Statistical Analyses

*Demographic and clinical characteristics.* Demographic and clinical characteristics between autistic adults and neurotypical controls were compared using independent t-tests for continuous variables and Chi-square tests for categorical variables. Statistical significance was set to $p < 0.05$.

*Between group comparisons.* Prior to inferential statistical analysis, a Shapiro-Wilk test was applied to assess the normality of all dependent variables. A total of 76.7% of the diffusion measures failed the test. We, therefore, implemented a one-way analysis of covariance (ANCOVA) with 5,000 permutations to assess between-group differences on each diffusion measure (i.e., FA, free water, or fwcFA) [45]. Each ANCOVA model consisted of group (ASD vs. NT) as the independent variable, a diffusion measure as the dependent variable, and age and sex as covariates. We introduced age and sex to ANCOVAs because our data comprised a wide age range, and sex has been shown to demonstrate a substantial impact on imaging measures in both autistic adults and neurotypical controls [46–48].

*Age effect.* Nonparametric partial correlation analyses with 5000 permutations were applied to examine the age effect on each diffusion metric separately for autistic adults and neurotypical controls [49]. Each correlation model consisted of age ( $X_{age}$) as the independent variable, a diffusion measure ( $Y$) as the dependent variable, and sex ( $Z_{sex}$) as the covariate following the formula below [50–52]:

$$Y = \alpha + X_{age} \cdot \beta + Z_{sex} \cdot \gamma + \epsilon \text{ (Eq. 1)}$$

where, $Y$ represents the diffusion measure of FA, free water, or fwcFA, $\beta$, and $\gamma$ stand for regression parameters, $\alpha$ is the intercept, and $\epsilon$ represents the random error. This analysis was repeated 756 times (378 diffusion measures $\times$ 2 groups = 756).

*Clinical correlation assessments.* Nonparametric partial correlation analyses with 5000 permutations were conducted to examine the relationship between dMRI measures that significantly differentiated autistic adults and neurotypical controls and clinical measures of ASD (AQ, SRS-2, RBS-R, and ADOS-2) [49].

*Correction for multiple comparisons.* ANCOVAs and nonparametric partial correlations were corrected for multiple comparisons using the false discovery rate (FDR) [53]. For each statistical approach, FDR was applied separately within each combination of diffusion measure and white/gray matter tissue category (e.g., FA in white matter, free water in gray matter). The *q* threshold was set at 0.05 [54]. Statistical analyses were conducted using SPSS version 29 (IBM SPSS Statistics, Armonk, NY, USA) and R version 4.2.2 (https://www.R-project.org).

## RESULTS

### Participants

Table 1 shows the demographic and clinical comparisons between autistic adults and neurotypical controls. Both groups were matched for age, sex, self-reported handedness, IQ scores, and total brain volume (all $p$s > 0.05). Autistic adults exhibited significantly higher scores on AQ, SRS-2, and RBS-R relative to neurotypical controls (all $p$s < 0.001).

### Between group comparisons in transcallosal white matter

Autistic adults exhibited FA reductions in 16 transcallosal tracts compared to neurotypical controls ($p_{raw}$ in Supplementary Table 1). However, only the ventral premotor cortex (PMv) and pre-supplementary motor area (preSMA) survived FDR correction (Fig. 1A and $p_{FDR}$ in Supplementary Table 1). Autistic adults also showed elevated free water in 24 transcallosal tracts relative to controls ($p_{raw}$ in Supplementary Table 2), where significance after FDR correction was maintained for 24 tracts (Fig. 1B and $p_{FDR}$ in Supplementary Table 2). This finding highlights widespread free water accumulations in the autistic brain ranging from frontal to occipital cortices. Autistic adults also exhibited reduced fwcFA for 6 transcallosal tracts in the occipital lobe, although all $p_{FDR}$ were non-significant (Fig. 1C and $p_{FDR}$ in Supplementary Table 3).

[Insert Fig. 1 about here]

## Between group comparisons in gray matter regions of interest (ROIs)

Autistic adults exhibited lower FA in 31 ROIs relative to neurotypical controls ($p_{raw}$ in Supplementary Table 4). However, only bilateral middle cingulate cortex (MCC) and left medial superior frontal gyrus (SFG_med) survived FDR correction (Fig. 2A and $p_{FDR}$ in Supplementary Table 4). Autistic adults demonstrated elevated free water in 15 gray matter ROIs ($p_{raw}$ in Supplementary Table 5), with only the left MCC survived FDR correction (Fig. 2B and $p_{FDR}$ in Supplementary Table 5). Additionally, autistic adults showed reduced fwcFA in 9 gray matter ROIs compared to controls ($p_{raw}$ in Supplementary Table 6). Similar to white matter, no between group difference survived FDR correction, suggesting negligible fwcFA differences in gray matter between autistic adults and controls (Fig. 2C and $p_{FDR}$ in Supplementary Table 6).

[Insert Fig. 2 about here]

## Age associated variations in dMRI measures are different between autistic adults and neurotypical controls

*Transcallosal white matter*. Figure 3A-3C show example scatterplots of dMRI measurements in superior parietal lobule (SPL) with respect to age progression. Strikingly different age-related patterns were observed for transcallosal tracts between autistic adults and neurotypical controls (Figs. 3D-F and Supplementary Table 7). While neurotypical controls exhibited age-related reductions across all ROIs for FA, this effect was only observed for inferior frontal gyrus pars opercularis (IFG_oper) in autistic adults (Fig. 3D). This widespread age effect extended similarly to free water, such that all regions exhibited age-related increases in free water for neurotypical controls, while there was no effect for any region in the autistic group (Fig. 3E). After free water correction, significant age-related reductions in fwcFA remained for 11 frontal transcallosal tracts in controls, but not for any white matter target in autistic adults (Fig. 3F).

[Insert Fig. 3 about here]

*Gray matter ROIs.* Largely disparate age-related patterns between autistic adults and neurotypical controls were also seen in gray matter (Supplementary Table 8). Age-related reductions in FA were observed for 27 ROIs in neurotypical controls but were absent in autistic adults. A positive association between age and free water was evident for 81 ROIs in neurotypical controls but was absent again in autistic adults. Upon free water correction, these age effects were no longer observed for both groups, suggesting that the age effect in gray matter was primarily driven by free water in controls.

#### Extended data inspection on age effect on white and gray matter between autistic adults and neurotypical controls

Extended data inspections were conducted on dMRI measures with respect to age progression to identify discrete age patterns in white and gray matter targets between autistic adults and neurotypical controls.

*Nonparametric partial correlation model.* We inspected $R^2$ and b derived from each nonparametric partial correlation model (Eq. 1). $R^2$ represents how well a diffusion measure fits the nonparametric linear regression model with respect to age progression (i.e., the goodness of fit) while b reflects the slope of the regression model (i.e., the direction of change). Specifically, a positive b indicates an increase in a diffusion measure with increasing age and vice versa (Montgomery et al., 2021). The distribution patterns of $R^2$ (Fig. 4) and b (Supplementary Fig. 1) were inspected for 32 transcallosal tracts and 94 gray matter ROIs in autistic adults and neurotypical controls, respectively.

For white matter, $R^2$ values of FA (Fig. 4A), free water (Fig. 4B), and fwcFA (Fig. 4C) were lower and sharply clustered leftward on the x-axis in the autistic group relative to the control group, demonstrating a lack of goodness of fit for the linear relationship between dMRI measurements and age in autistic adults. Conversely, $R^2$ values were more positive with greater spread across the x-axis in neurotypical controls, demonstrating greater linear relationships with advancing age across multiple cortices. Additionally, FA and fwcFA of the frontal transcallosal tracts exhibited the largest between-group difference on $R^2$ mean values with respect to age progression (black arrows in Figs. 4A and 4C), which suggests a considerable discrepancy in FA and fwcFA for the frontal area between autistic adults and neurotypical controls.

[Insert Fig. 4 about here]

For gray matter, autistic adults also showed lower $R^2$ values in FA and free water and sharply leftward distributions on the x-axis relative to neurotypical controls (Figs. 4D and 4E). However, when examining fwcFA, $R^2$ values of both autistic adults and controls were centered around zero, suggesting a lack of goodness of linear fit for both groups respective to age progression (Fig. 4F).

Supplementary Fig. 1 shows dispersion maps of b in autistic adults and neurotypical controls. For controls, negative bs were found in FA and fwcFA, while positive values were found in free water of the white and gray matter, suggesting that FA and fwcFA decrease while free water increase of the whole brain with advancing age. In contrast, b mean values were more centered around 0 on the x axis in autistic adults compared to neurotypical controls, suggesting a less prominent age-related directional change in dMRI measures in autistic adults.

*Nonlinear regression model.* A nonlinear regression model was applied to further explore age effect for both groups because the lack of fit for linear models in quantifying age-associated variations in white and gray matter for autistic adults (Supplementary Tables 9 and 10) and previous studies have identified quadratic relationships in brain variations with age in young autistic individuals (Zielinski et al 2014; Andrews et al. 2021). Our nonparametric quadratic model consisted of age and age-squared as independent variables, a diffusion measure as the dependent variable, and sex as the covariate. Across diffusion measures, brain regions, and groups, fwcFA of 4 transcallosal tracts showed significance with age-squared. These transcallosal tracts were paracentral lobule (PCL: $p = .014$) for autistic adults and inferior occipital gyrus (IOG: $p = .011$), middle occipital gyrus (MOG: $p = .009$), and superior occipital gyrus (SOG: $p = .016$) for neurotypical controls. This finding suggests that quadratic age effects were not robust in either group, which reiterates our approach of using nonparametric partial correlation model to examine the age effect for both autistic adults and controls.

## Clinical correlations

Autistic adults demonstrated a global elevation of free water in 24 transcallosal tracts relative to neurotypical controls (Fig. 1B and Supplementary Table 2). Nonparametric partial correlation analysis was applied subsequently to assess the relationship of free water in these transcallosal tracts to clinical measurements of ASD, including AQ, SRS-2, RBS-R, and ADOS-2 (Supplementary Table 11). After FDR correction, no significant correlations were found between free water of 24 transcallosal tracts and each of the SRS-2 *t* score, RBS-R total score, or ADOS-2 total raw score for autistic adults. Greater AQ total raw score was associated with increased free water in the inferior frontal gyrus pars orbitalis (r = 0.438, $p_{FDR} = 0.046$) and lateral orbital gyrus (r = 0.434, $p_{FDR} = 0.046$) in autistic adults.

### DISCUSSION

Autism spectrum disorder (ASD) is a lifelong condition [1–4]; however, little is known about age-associated microstructural deviations across the whole brain in autistic adults aged > 30 years. The present work is the first to quantify the microscopic architecture of transcallosal tracts and gray matter ROIs in autistic adults and the age-associated variations in dMRI measures between autistic adults and neurotypical controls. We identified two novel findings. First, autistic adults demonstrated increased free water across most transcallosal tracts (Fig. 1B) but not gray matter ROIs (Fig. 2B) relative to neurotypical controls. Second, in control participants, age was correlated with FA reductions and free water increases in both white (Figs. 3D and 3E and Supplementary Table 7) and gray matter regions (Supplementary Table 8). However, this brain aging profile was absent in autistic adults. Our findings suggested differential microstructural variations in white and gray matter in autistic adults, and age-associated deviations in dMRI measures present more heterogeneous profiles in autistic adults.

## Globally elevated free water in transcallosal white matter in autistic adults

Negligible FA and fwcFA differences between the two groups highlighted indifferent white matter microstructural features in autistic adults relative to neurotypical controls (Figs. 1A and 1C and Supplementary Tables 1 and 3). This finding is consistent with previous studies in middle and old aged autistic adults, which have shown no differences in FA, MD, and RD in ASD relative to controls [28, 29].

Globally elevated free water was identified across 24 transcallosal tracts in autistic adults (Fig. 1B and Supplementary Table 2). This finding is consistent with a previous study, which has identified increased isotropic volume fraction (ISOVF) in commissural tracts in autistic adults using neurite orientation and dispersion imaging (NODDI) [55]. Higher ISOVF has been associated with increased extracellular free water in NODDI despite a very recent study suggesting a possible overestimation of the free water volume fraction in white matter [56]. Elevated free water in extracellular space may reflect neuroinflammation and axonal degeneration [57–60]. Despite the challenge of identifying precise pathophysiological substrates through dMRI, multiple converging lines of evidence support that amplified neuroinflammation may underlie the global increase of free water in autistic adults. For example, postmortem studies have identified sustained activation of astroglia and microglia and elevated proinflammatory cytokine and chemokine in autistic individuals [21–25]. Preclinical research has also demonstrated increased permeability of the blood-brain barrier in animal models of ASD, which leads to heightened translocation of inflammatory mediators and immune cells from peripheral blood to the brain [61]. Studies of Alzheimer's disease have previously shown that neuroinflammation typically precedes microscopic tissue damage (e.g., demyelination or axonal atrophy), particularly during the prodromal stage of disease [15, 62, 63]. Globally elevated free water in transcallosal tracts may reflect altered immune responses of ASD [21, 24, 64], prodromal neuroinflammation prior to more pronounced stages of neurodegeneration, or both. Future research is warranted to monitor free water and dMRI measure changes in autistic adults longitudinally to parse the complexity of aging-associated white matter deviations in ASD.

## Negligible gray matter variations in autistic adults relative to neurotypical controls

Unlike white matter, the left MCC was the only ROI exhibiting elevated free water in autistic adults (Fig. 2B and Supplementary Table 5). Some previous studies have shown reductions in gyrification and cortical folding in the frontal, temporal, and parietal areas in autistic adults [31] while others have reported no differences in age-related volume loss and cortical thinning between the two groups [31]. Our finding of increased free water in the left MCC suggested a more localized neuronal variation in autistic adults. Additionally, autistic adults demonstrated no differences in fwcFA values across all gray matter ROIs relative to controls, further supporting indifferent microstructural characteristics in gray matter (e.g., neuron cell bodies, synapses, and dendrites) between the two groups (Fig. 2C and

Supplementary Table 6). Combined with findings in white matter, our study suggested that differential microscopic integrity between white and gray matter in autistic adults.

## Age-associated variations in dMRI measures are different between autistic adults and neurotypical controls

Despite regional variations, several studies on lifespan changes in white matter have found accelerated FA reduction and free water increase as individuals age [12, 13, 65]. This typical brain aging profile is associated with multiple neurobiological processes, including myelin sheaths ballooning [66], reductions in myelin and synapses [67, 68], or myelinated fiber shrinkage [67, 69]. Consistent with previous observations, control participants in our study demonstrated similar age associated FA and free water patterns in both white and gray matter (Fig. 3 and supplementary Tables 7 and 8). Notably, after free water correction, age-associated fwcFA reductions only remained for frontal transcallosal tracts and were completely absent in gray matter in controls, highlighting elevated free water in extracellular space in driving the age effect in FA, particularly for the gray matter, rather than alterations in tissue microstructure.

FA and fwcFA in the frontal transcallosal tracts also exhibited the greatest between-group discrepancies on $R^2$ mean values (black arrows in Figs. 4A and 4C). This finding was primarily driven by FA and fwcFA reductions in the frontal transcallosal tracts in controls and more variable FA and fwcFA distributions with respect to age progression in autistic adults (Figs. 3A and 3B). Age-associated dMRI metrics pattern in controls echo the “last in, first out” hypothesis, stating that neocortices, particularly the frontal and prefrontal areas, developed the latest are the first to be affected by aging [70]. In contrast, distinct age associated dMRI patterns found in neurotypical controls were largely absent in autistic adults (Figs. 3D and 3E and Supplementary Tables 7 and 8). Empirical studies exploring cognitive and brain morphological differences in middle and old aged autistic adults have proposed three discrete theories associated with aging [71, 72]. The safeguard theory hypothesizes the existence of some protective neurobiological mechanisms against brain degeneration in autistic adults [31]. In contrast, the double jeopardy theory proposes a cumulative effect of aging on pre-existing brain deviations in ASD, leading to accelerated decline as individuals age [27–29, 73]. Lastly, the parallel development theory supports typical aging trajectories in middle and old aged autistic adults [71, 74, 75]. Our results appeared to support highly heterogeneous white and gray matter variations with the progression of age in ASD. Future large-cohort studies are needed to identify subgroups of autistic individuals who demonstrate accelerated, similar, or slower brain aging profiles relative to neurotypical controls.

## Free water as a critical dMRI metric for quantifying microstructural variations in autistic adults

The assessment of free water and free water-corrected dMRI measures has been limited in studies of ASD [26, 27]. Previous research in autistic children and young adults has consistently shown reduced FA and increased MD and RD in the corpus callosum cross-sectionally [76–78] and longitudinally [79], supporting the notion of reduced long-distance and inter-hemispheric connectivity in ASD [9, 10].

Although contradictory findings have been reported [80–83], discrepancies were mainly attributed to differences in scanner type, scan sequence, data analytical procedures (e.g., voxel-, ROI-, or tractography-based methods), demographic characteristics of participants, or ASD heterogeneity. The effect of extracellular free water on biasing dMRI metrics has been largely overlooked when interpreting inconsistent findings [17–19]. Our study underscores the importance of quantifying free water in dMRI assessment as we have found globally evaluated free water in 24 transcallosal white matter tracts in autistic adults. Our study also demonstrated free water as a sensitive marker for aging in neurotypical controls as it has shown significant correlations with age in white and gray matter [17, 20] .

## Free water associated clinical correlations

Elevated free water in the IFG_oper and LOG tracts was associated with higher AQ total raw scores in autistic adults (Supplementary Table 11). Both transcallosal tracts were commonly implicated in imaging studies of ASD. As part of the mirror neuron system, the inferior frontal gyrus pars orbitalis is highly involved in social-emotional regulation, language processing, and communication in ASD [84]. The lateral orbital gyrus is also a key area for emotional control following the reward and non-reward tradeoffs [85]. Free water associated clinical correlations in autistic adults reported here may provide important indices of neurophysiological mechanisms contributing to autistic traits.

## LIMITATIONS AND FUTURE DIRECTIONS

Our findings should be considered in the context of several limitations. First, our study only recruited cognitively capable autistic adults. Individuals with comorbid intellectual disability and dementia would need to be included in future studies to allow a comprehensive understanding of aging-associated white and gray matter deviations and identify confounding variables that may accelerate pathological aging conditions in ASD. Second, our study involved a cross-sectional design, while inferences about aging-related microstructural changes in white and gray matter can only be quantified through longitudinal studies [86]. Given the lack of age effect on dMRI measures in autistic adults, future longitudinal studies with a large cohort of autistic adults aged > 30 years are urgently needed in parsing discrete brain aging profiles in ASD. This line of research would also benefit from cluster analysis to identify subgroups of autistic adults who are more vulnerable to developing neurodegenerative diseases versus individuals who are protected from pathological aging conditions [72]. Lastly, long-term psychotropic medication exposure alters microscopic configurations of the brain in individuals with schizophrenia and bipolar disorder [87, 88]. Future studies are warranted to quantify long-term medication effects on brain changes in autistic adults and determine whether this effect would slow down or accelerate pathological aging conditions in ASD.

## CONCLUSION

Our study is the first to comprehensively quantify free water and free water corrected dMRI measures in white and gray matter in autistic adults and matched neurotypical controls. Globally elevated free water in 24 transcallosal white matter tracts was found in autistic adults. Age-associated FA reductions and free water increases were identified in white and gray matter ROIs in controls but were largely absent in autistic adults. These findings require replication in larger samples, especially the extent to which free water biases single tensor dMRI measures in studies of ASD. Future longitudinal research is needed to monitor free water and free water corrected dMRI measure changes across the adult lifespan in ASD.

## Declarations

#### Acknowledgments

We extend our sincere thanks to all the autistic adults and control individuals who participated in this study. We are grateful to CARD directors Ann-Marie Orlando, Ph.D., at the University of Florida, Teresa Daly, Ph.D., at the University of Central Florida, and Beth Boone, Ph.D., at the University of South Florida, and the SPARK Research Match Team for their support in identifying and recruiting autistic adults.

#### Ethics approval

All procedures involved in this study were approved by the Institutional Review Board (IRB) at the University of Florida following the Declaration of Helsinki. The IRB number is 202100659, with an approval date of July 26, 2022.

#### Data availability

All data are available from the corresponding author upon reasonable request.

#### Competing interests

No claimed competing interest.

#### Consent for publication

All authors have read and approved the submission.

#### Funding

Dr. Zheng Wang receives funding from the National Institute on Aging (R21AG 065621 and R01AG086493), the National Institute of Neurological Disorders and Stroke (R01NS121120), and the University of Florida APK Research Investment Grants Award. Dr. Stephen Coombes receives funding from the National Institute on Aging (R01AG080468 and P30AG066506).

#### Author contributions

ZW and SC conceptualized and designed the study. DJS screened, recruited, and consented participants. DJS collected all demographic and dMRI data. AMO and RAR confirmed the diagnosis for autistic adults. DJS entered demographic and clinical data into the database. YSS and SC performed brain imaging data analysis. YSS and JW conducted statistical analyses. YSS and JW prepared graphic presentations and tables of the results. YSS, DC, JW, BW, SC, and ZW interpreted the results. YSS, DC and ZW drafted the manuscript. All authors edited the manuscript and approved the final version of the manuscript.

## Figures

**Figure 1.**

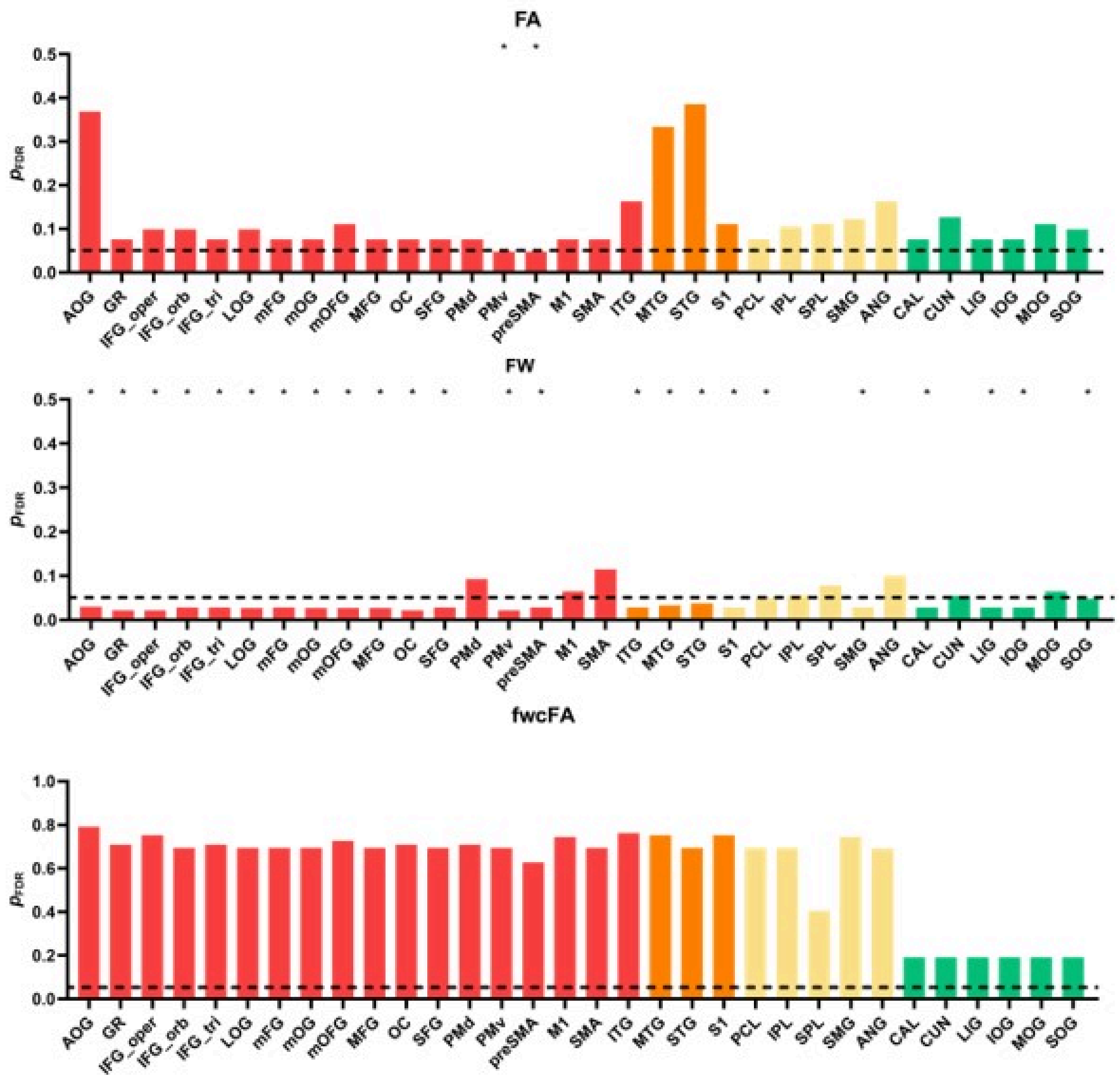

### Figure 1

ANCOVA results of FA (A), free water (B), and fwcFA (C) of 32 transcallosal tracts in the frontal (red), temporal (orange), parietal (yellow), and occipital (green) areas. The dashed horizontal line represents a *q-value threshold* of 0.05 for FDR correction. Bars below the dashed line (also labeled with * on top of the bars) indicate significant between-group differences.

**Figure 2.**

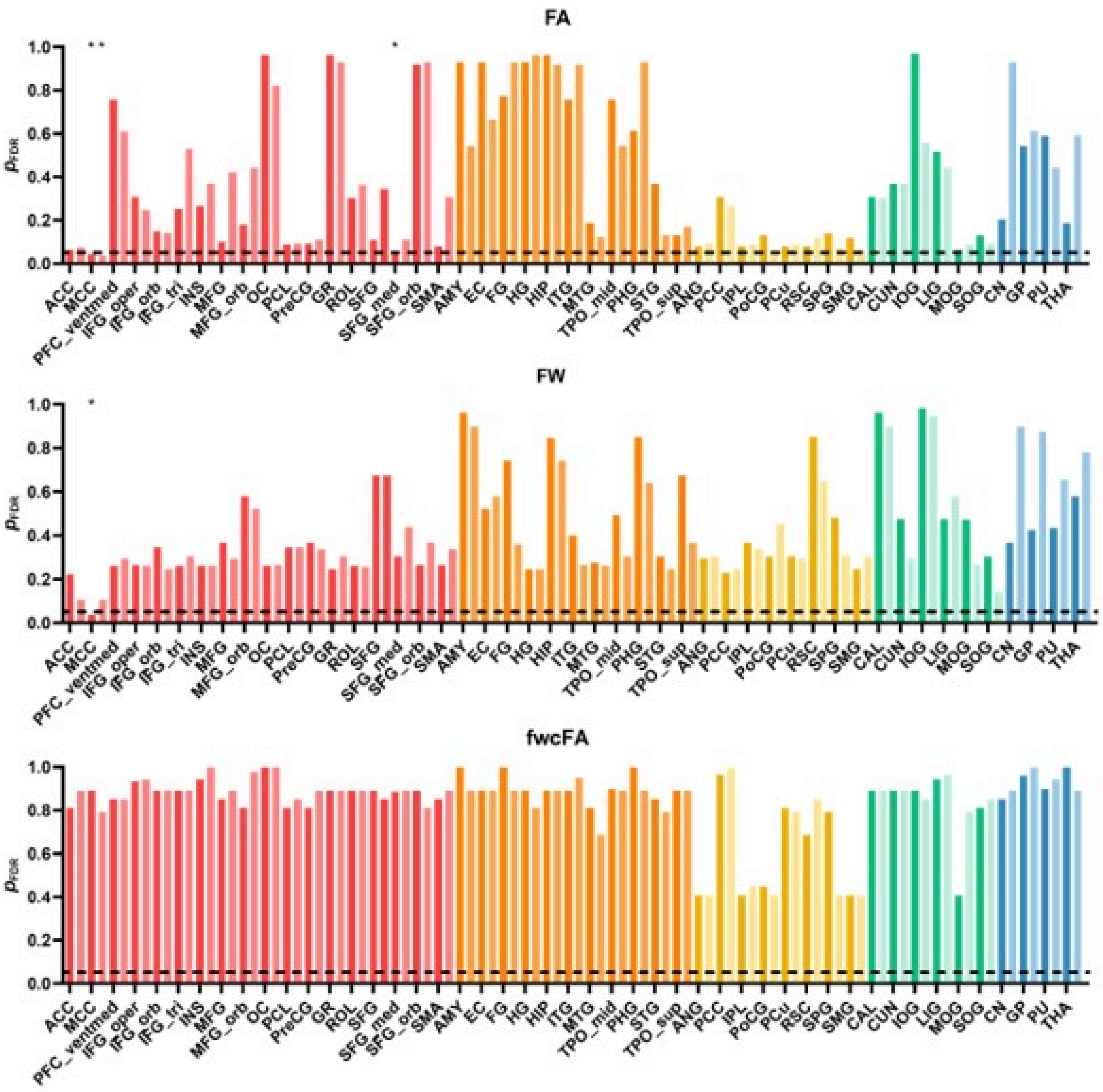

### Figure 2

ANCOVA results of FA (A), free water (B), and fwcFA (C) of 94 gray matter ROIs (B) in the frontal (red), temporal (orange), parietal (yellow), occipital (green), and subcortical (blue) areas. **Gray matter ROIs within the left and right hemispheres are coded in dark and light colors, respectively.** The dashed horizontal line represents a *q-value threshold* of 0.05 for FDR correction. Bars below the dashed line (also labeled with * on top of the bars) indicate significant between-group differences.

**Figure 3.**

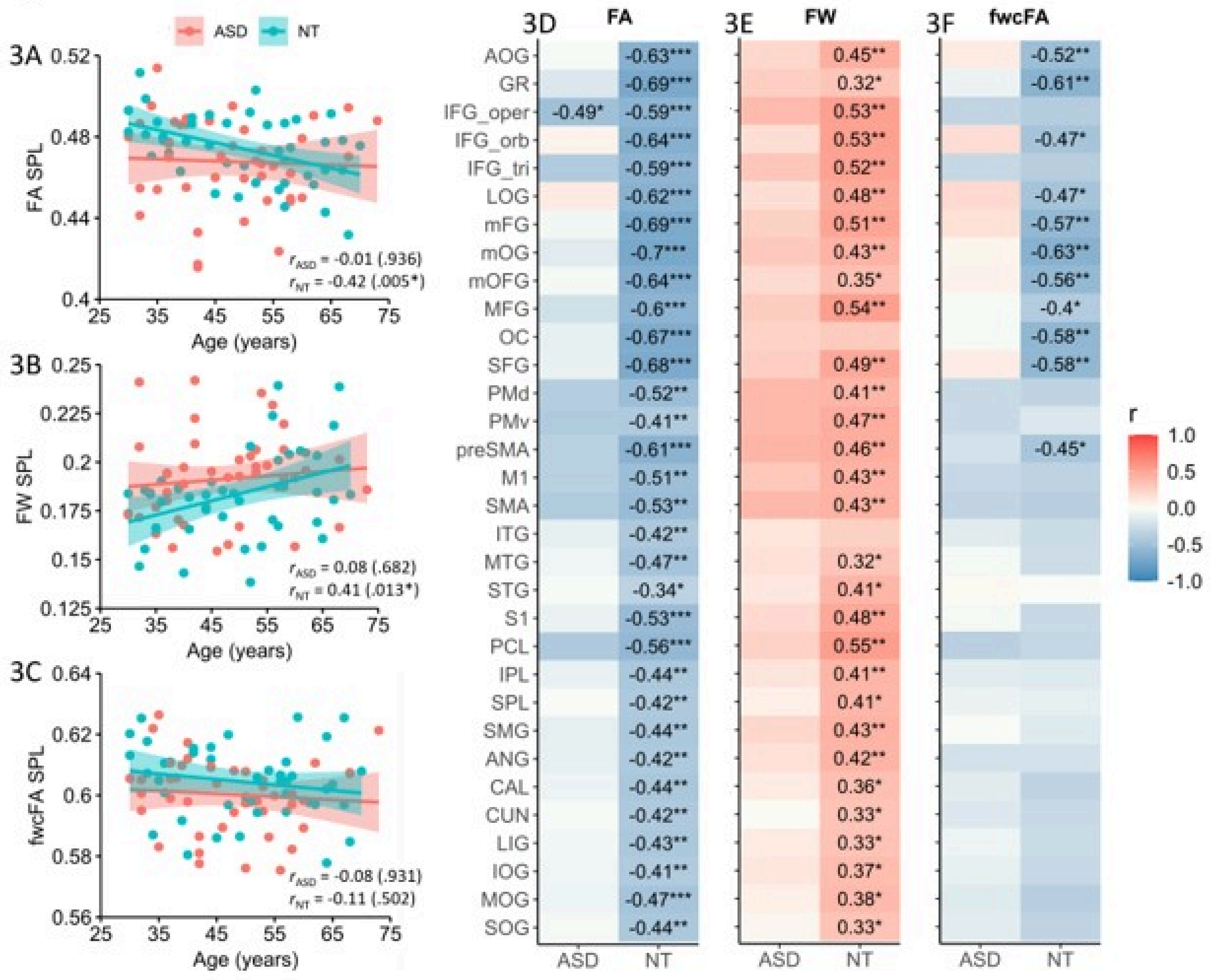

### Figure 3

Nonparametric partial correlation results of age on dMRI metrics of transcallosal tracts. Scatterplots of FA (A), free water (B) and fwcFA (C) in superior parietal lobule (SPL) show relationship between dMRI measures and age in autistic adults (rose red) and neurotypical controls (sky blue). Parentheses indicate $p_{FDR}$. Age associated correlation results for FA (D), free water (E), and fwcFA (F) are arranged from the left to the right. Color gradient ranges from dark blue to dark red, representing r values from -1 (negatively correlated) to 1 (positively correlated). Only r values survived FDR correction are labeled. Statistical significance is labeled at three levels: *p< 0.05, **p<0.01, ***p<0.001. Refer to Supplementary Table 7 for all nonparametric partial correlation results for white matter.

**Figure 4.**

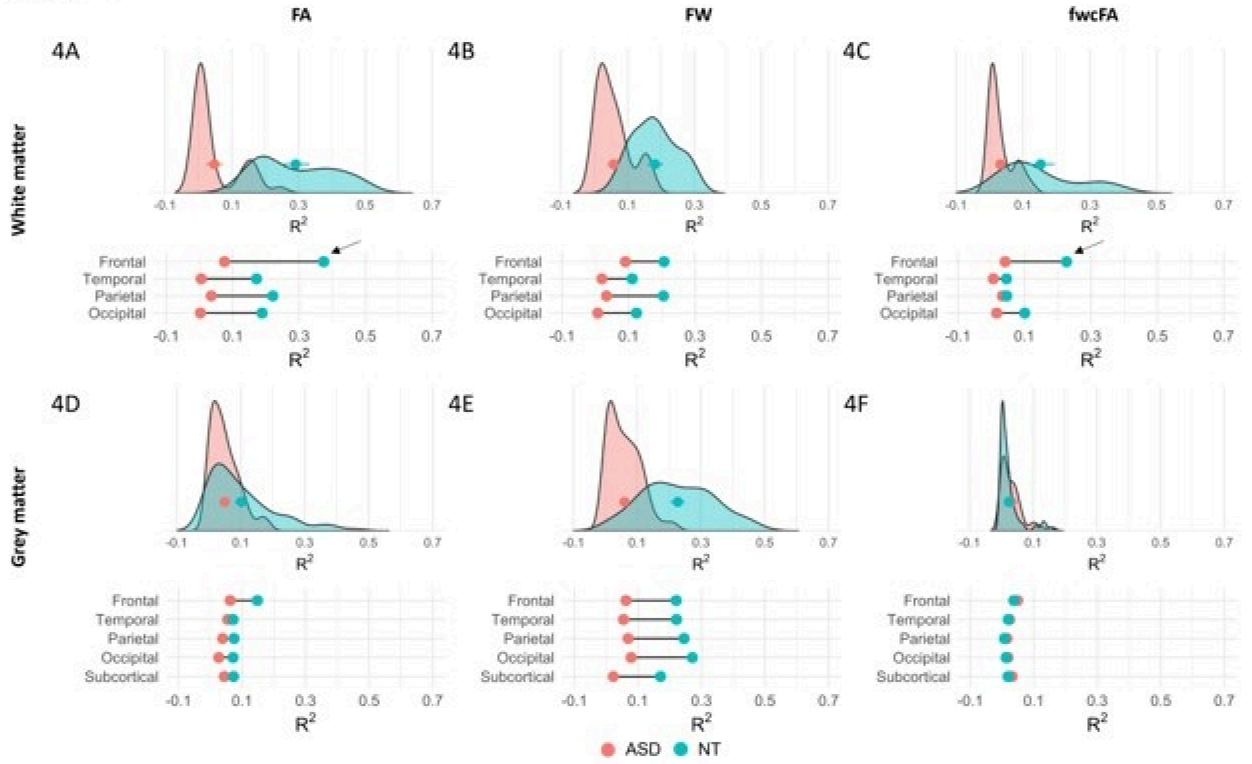

**Figure 4.** $R^2$ dispersion plots for 32 transcallosal tracts (top panel) and 94 gray matter ROIs (bottom panel) of autistic adults (rose red) and neurotypical controls (sky blue). Dispersion plots for FA (A), free water (B), and fwcFA (C) are arranged from the left to the right. The ⬤ that sits in the middle of each dispersion plot represents the [Mean $\pm$ SE] of $R^2$ value for each autism or control group. The [Mean]s of $R^2$ values derived from each of the frontal, temporal, parietal, and occipital cortices are displayed at the bottom panel of each dispersion plot cluster and labeled by ⬤. The lines connecting these red and blue dots represent the $R^2$ mean difference between the autism and control groups.

#### Figure 4

See image above for figure legend

### Supplementary Files

This is a list of supplementary files associated with this preprint. Click to download.

- SupplementaryMaterials.docx
